# Evaluating PSA Density as a Predictor of Biochemical Failure after Radical Prostatectomy: Results of a Prospective Study after a Median Follow-Up of 36 Months

**DOI:** 10.1155/2013/984951

**Published:** 2013-05-16

**Authors:** Stavros Sfoungaristos, Petros Perimenis

**Affiliations:** Urology Department, Patras University Hospital, Building A, 4th Floor, Rion, 26500 Patras, Greece

## Abstract

*Purpose*. To evaluate the predictive ability of PSA density for biochemical relapse after radical prostatectomy in patients operated for clinically localized disease and to compare its predictive strength with preoperative PSA and Gleason score. *Patients and Methods*. The study evaluated 244 patients with localized disease who underwent an open retropubic radical prostatectomy between February 2007 and April 2011. PSA was measured every 3 months after surgery with a mean follow-up period of 36 months. Two consecutive rises >0.2 ng/mL were considered as biochemical relapse. *Results*. Biochemical recurrence was observed in 71 (29.1%). A great correlation was found between relapse and PSA (*P* = 0.005), PSA density (*P* = 0.002), Gleason score (*P* = 0.015), pathological stage (*P* = 0.001), positive surgical margins (*P* = 0.021), and invasion of seminal vesicles (*P* < 0.001) and lymph nodes (*P* < 0.001). We also found that PSA density was associated with adverse pathological findings. In univariate and multivariate analysis both PSA (*P* = 0.006) and PSA density (*P* = 0.009) were found to be significant predictors for relapse in contrast to tumor grade. *Conclusion*. PSA density is a valuable parameter in estimating the danger of biochemical failure and it may increase predictive potential through the incorporation in preoperative nomograms.

## 1. Introduction

The implementation of PSA in the everyday clinical practice has revolutionized prostate cancer screening leading to early detection of the disease and improved survival outcomes [1]. Concomitantly, earlier detection has led to a stage migration and a significant number of the patients who are operated are suffering from low volume even insignificant disease. On the other hand, a great proportion of patients whom the disease is classified preoperatively as organ confined found to have a greater stage and grade prostate cancer, determined after surgical specimen analysis, and such results can influence prognosis and survival [2].

The optimal therapy for localized prostate cancer is still controversial with radical prostatectomy to be the main therapeutic option. The main tools used to estimate the preoperative risk for adverse pathological findings after surgery and the possibility for PSA recurrence after radical treatment are PSA, Gleason score, and clinical stage [3, 4]. However, the rate of biochemical recurrence after surgery is estimated to be around 17% and this rate is reaching 33% in high risk patients [5, 6]. Therefore, improved tools to predict pathological stage and biochemical recurrence are required.

Although PSA density has a significant and established role in prostate cancer screening by increasing the diagnostic value of PSA [7], its role as a predictor of biochemical relapse after radical prostatectomy has not been identified and limited number of studies have retrieved with this subject.

The aim of the present study was to evaluate PSA density as a predictor of biochemical recurrence in patients undergoing a radical prostatectomy for clinically localized disease. Furthermore, a comparison was made between the latter, PSA, and Gleason summary to identify their predictive strength.

## 2. Patients and Methods

After we obtained an approval from the Ethics committee of our institution, we conducted a prospective study of 256 consecutive patients who underwent an open retropubic radical prostatectomy. All patients suffered from clinically localized prostate cancer, as it had been defined from the findings of preoperative digital rectal examination, transrectal ultrasound, and in some cases (29 patients) of magnetic resonance imaging, and they had >10 years of life expectance. An abdomen and pelvic computer tomography was asked from patients with PSA >20 ng/mL and/or Gleason score ≥7, and/or positive digital rectal examination while a bone scintigraphy was performed in cases with PSA >20 ng/mL, Gleason score >7 and/or clinical stage ≥T2c. The diagnosis of the disease was established by positive biopsy results. Patients with any clinical suspicion of locally advanced prostate cancer were excluded from the study. None of the patients had received neoadjuvant or adjuvant hormone therapy and/or radiotherapy. The study period was from February 2007 to April 2011.

All surgical procedures were made by a single surgeon. In all cases, a standard surgical procedure was followed as it has been described by Walsh and Donker [8], including the removal of the prostate and seminal vesicles. An extended pelvic lymph node dissection (including the external iliac, obturator, hypogastric, presacral, and common iliac lymph nodes until the level of the aortic bifurcation) was not routinely performed and was preserved for those (153 patients) with a greater than 7% risk for lymph node invasion based on the nomograms of Partin [4]. The caudal limit of the lymphadenectomy was the femoral canal. The removed connective and lymphatic tissue was fixed in neutral buffered 4% formaldehyde for 24 hours and then placed in acetone to dissolve the fatty tissue.

The surgical specimen was examined of our institution pathologists and a histological report concerning the presence of organ confined disease, the presence of positive surgical margins, and the pathological grade and stage was obtained. Any extension of tumor outside of the prostatic capsule in the periprostatic fat was considered as advanced disease while the infiltration of the capsule without penetration was considered as localized disease. The 2009 TNM (tumour node metastasis) classification was used to define the pathological stage.

Follow-up protocol was consisted by visits every 3 months in order to evaluate the levels of serum PSA. A digital rectal examination was performed in all patients in every visit. Biochemical relapse was defined as 2 consecutive values >0.2 ng/mL, 2 weeks apart. We have to point that no adjuvant therapeutic mode was applied in all cases, even in the cases with advanced disease, until the time of biochemical failure. The median followup was 36 months.

The main endpoint of the study was to evaluate the role of PSA density in predicting biochemical recurrence in patients who were operated for clinically localized prostate cancer. Secondary endpoints were the correlation of PSA density with findings of pathological adverse events, in terms of positive surgical margins, extracapsular disease, seminal vesicles invasion and lymph nodes invasion, and to compare PSA density, PSA and Gleason score potential in predicting biochemical recurrence.

PSA density was calculated preoperatively during the biopsy procedure by using transrectal ultrasound by dividing the maximum preoperative PSA value and prostate volume. The latter was calculated based on the ellipse dimension theory formula [9] (*D*1 × *D*2 × *D*3 × *pi*/6), where *D*1 is the maximum transverse diameter, *D*2 is the maximum anteroposterior diameter, *D*3 is the maximum longitudinal diameter, and *pi* is a mathematical constant approximately equal to 3.14. All transrectal ultrasound procedures and calculations were made by a single operator.

Statistical analysis was performed by using SPSS version 17 (SPSS Inc., Chicago, IL, USA). Descriptive statistics are presented as the mean ± standard deviation and interquartile range for continuous variables and as the absolute and percent frequency for categorical variables. The numerical variables normality condition was studied by using Kolmogorov-Smirnov test. None of the variables had a normal distribution and thus Mann-Whitney *U* test was used to compare means between groups. Chi-square *χ* ^2^ test was used for categorical variables. A univariate analysis was performed to identify the predictive significance of PSA density, preoperative PSA, and preoperative Gleason score in prediction of biochemical recurrence. A multivariate analysis was performed then for the variables identified as statistically important in univariate analysis, using logistic regression. Pearson's correlation was used to evaluate the association of PSA density with the presence of positive surgical margins, extracapsular disease, invasion of seminal vesicles and lymph nodes, and biochemical recurrence. All tests were 2-tailed with *P* < 0.05 to be considered as a statistically significant value.

## 3. Results

Twelve patients were excluded due to insufficient data (lost during the followup). Finally, 244 patients entered the analysis providing a margin error of the study estimated to 6.02%. The characteristics of the study cohort are summarized in Table 1. As we can see in Table 2, 71 patients (29.1%) had biochemical failure during followup. There was a statistically significant correlation between preoperative PSA (*P* = 0.005), PSA density (*P* = 0.002), and Gleason score (*P* = 0.015) and disease recurrence. PSA relapse was also significantly correlated with pathological stage (*P* = 0.001), the presence of positive surgical margins (*P* = 0.021), and invasion of seminal vesicles (*P* < 0.001) and lymph nodes (*P* < 0.001). In addition, we found that levels of PSA density are strongly associated with the adverse pathological findings (Table 3). In particular, PSA density was significantly correlated to extracapsular disease (*P* < 0.001), seminal vesicles invasion (*P* = 0.009), and the possibility for biochemical relapse (*P* = 0.040). The analysis failed to reveal any association with positive surgical margins and lymph node disease.

A univariate and multivariate analysis was conducted to explore and compare the ability of PSA, PSA density and Gleason score in prediction of biochemical failure (Table 4). Both PSA and PSA density found to be strong predictors for that in contrast with tumor grade. PSA had (*P* = 0.006) marginal predominance compared to PSA density (*P* = 0.009).

## 4. Discussion

The incidence of prostate cancer has tremendously increased after the introduction of PSA in disease screening and a greater number of patients will be operated in order to radically excise the tumor. Given that a significant proportion of these patients will relapse after surgery, preoperative parameters predictive of tumor status and the potential for biochemical failure are required.

PSA density is mainly used to distinguish “benign” increases of PSA from “malignant” ones especially in cases belonging in the grey zone, meaning PSA rises between 4 and 10 ng/mL. Additionally, previous reports have revealed the valuable role of PSA density in estimating the possibility of adverse pathological findings after radical prostatectomy [10–16].

Kundu et al. [15] reported that PSA density can be used as an adjunct in predicting insignificant cancer and outcomes after local therapy. Similar to our results, the authors found that PSA density was highly associated with biochemical recurrence postoperatively. However, no comparison between the elative accuracy of PSA and its density was made. Similar results were revealed by the study of Freedland et al. [12]. Pathological PSA density was an independent predictor of biochemical failure providing that if PSA density combined with biopsy Gleason score offers better risk stratification for biochemical failure than a combination of PSA and biopsy Gleason score. PSAD was also found to be an independent predictor of biochemical recurrence in the study of Jones et al. [14]. However, a comparison of areas under the curve yielded no significant difference between PSA and PSA density in predicting biochemical recurrence following radical prostatectomy. PSA density either calculated preoperatively or postoperatively using pathological organ volume or weight was also found to be correlated with PSA relapse in the study of Brassell et al. [16]. However, PSA was significantly better in predicting biochemical recurrence than PSA density, irrespective of the way it was calculated. Magheli et al. [17] found that PSA density is highly associated with biochemical free survival following radical prostatectomy. In lower grade prostate cancers, meaning Gleason score ≤6, PSA density is significantly more accurate for predicting biochemical failure compared to total PSA.

In the present study we showed that PSA density significantly correlates with adverse pathological features after radical prostatectomy and with the possibility of PSA relapse, as well. In addition, it was found that PSA density was a strong preoperative predictor, though slightly inferior to PSA, for biochemical failure after radical therapy. Given that PSA relapse after radical surgery is seriously influencing the prognosis and quality of life due to additional therapies, preoperative estimation of patients with increased potential for such a failure is of great clinical importance since the suggested treatment protocols and patient information may be positively altered. Our findings suggest that PSA density deserves a place in preoperative nomograms since it may increase the prognostic ability of the current ones. In the continuous effort of urological community and researchers to better define prostate cancer nature and biological aggressiveness, PSA density seems to be significant for estimating the potential outcome after radical prostatectomy.

## 5. Conclusion

Prediction of biochemical failure after radical prostatectomy is of great importance since it represents an adverse factor for prognosis and survival. The most commonly used nomograms are based upon preoperative stage, PSA and Gleason score. Based on our results, PSA density is a significant predictor and its implementation in nomograms may increase their accuracy in estimating postoperative PSA relapse.

## Figures and Tables

**Table 1:** Study cohort characteristics.

Characteristics		Kolmogorov-Smirnov
Number of patients, *n *	244	
Age (years)		
Mean ± std, IQR	66.2 ± 6.49, 10	*P* < 0.001
PSA (ng/mL)		
Mean ± std, IQR	8.85 ± 3.66, 4.79	*P* < 0.001
PSA density (ng/mL^2^)		
Mean ± std, IQR	0.34 ± 0.38, 0.19	*P* < 0.001
Preoperative Gleason score, *n* (%)		
≤6	87 (35.7)	
7	118 (48.4)	
≥8	39 (16.0)	
Pathological Gleason score, *n* (%)		
≤6	86 (35.2)	
7	112 (45.9)	
≥8	46 (18.9)	
Clinical stage, *n* (%)		
T1c	181 (74.2)	
T2a	45 (18.4)	
T2b	16 (6.6)	
T2c	2 (0.8)	
Pathological stage, *n* (%)		
T2a	25 (10.2)	
T2b	8 (3.3)	
T2c	142 (58.2)	
T3a	47 (19.3)	
T3b	22 (9.0)	
Extracapsular disease, *n* (%)		
No	175 (71.7)	
Yes	69 (28.3)	
Surgical margins, *n* (%)		
Negative	167 (68.4)	
Positive	77 (31.6)	
Seminal vesicles invasion, *n* (%)		
No	222 (91.0)	
Yes	22 (9.0)	
Lymph nodes invasion, *n* (%)		
Not performed	91 (37.3)	
No	122 (50.0)	
Yes	31 (12.7)	
Biochemical failure, *n* (%)		
No	173 (70.9)	
Yes	71 (29.1)	

Std: standard deviation; IQR: interquartile range.

**Table 2:** Clinical and pathological characteristics of study cohort depending on the presence of biochemical recurrence.

	No recurrence	Recurrence	*P *
Number of patients, (%)	173 (70.9)	71 (29.1)	
Age (years), mean ± std, IQR	66.3 ± 6.56, 9	65.9 ± 6.36, 11	0.527^†^
PSA (ng/mL), mean ± std, IQR	8.37 ± 3.23, 4.29	9.99 ± 4.35, 5.20	0.005^†∗^
PSA density (ng/mL^2^), mean ± std, IQR	0.29 ± 0.25, 0.18	0.46 ± 0.57, 0.27	0.002^†∗^
Preoperative Gleason score, *n* (%)			0.015^‡∗^
≤6	71 (41.0)	16 (22.5)	
7	79 (45.7)	39 (54.9)	
≥8	23 (13.3)	16 (22.5)	
Pathological Gleason score, *n* (%)			0.423^‡^
≤6	65 (37.6)	21 (29.6)	
7	78 (45.1)	34 (47.9)	
≥8	30 (17.3)	16 (22.5)	
Clinical stage, *n* (%)			0.201^‡^
T1c	134 (77.5)	47 (66.2)	
T2a	29 (16.8)	16 (22.5)	
T2b	8 (4.6)	8 (11.3)	
T2c	2 (1.2)	0 (0)	
Pathological stage, *n* (%)			0.001^‡∗^
T2a	19 (11.0)	6 (8.45)	
T2b	7 (4.05)	1 (1.41)	
T2c	104 (60.1)	38 (53.5)	
T3a	36 (20.8)	11 (15.5)	
T3b	7 (4.05)	15 (21.1)	
Extracapsular disease, *n* (%)			0.064^‡^
No	130 (75.1)	45 (63.4)	
Yes	43 (24.9)	26 (36.6)	
Surgical margins, *n* (%)			0.021^‡∗^
Negative	126 (72.8)	41 (57.7)	
Positive	47 (27.2)	30 (42.3)	
Seminal vesicles invasion, *n* (%)			<0.001^‡∗^
No	166 (96.0)	56 (78.9)	
Yes	7 (4.05)	15 (21.1)	
Lymph nodes invasion, *n* (%)			<0.001^‡∗^
No	83 (100.0)	39 (55.7)	
Yes	0 (0)	31 (44.3)	

^†^Mann-Whitney *U* test, ^‡^chi-square test, *statistically significant, std: standard deviation, and IQR: interquartile range.

**Table 3:** Correlation of PSA density to adverse pathological findings and biochemical recurrence.

	PSM	ECD	SVI	LNI	BCR
Mean ± std	0.32 ± 0.47	0.28 ± 0.45	0.09 ± 0.29	0.40 ± 0.45	0.29 ± 0.46
*P* (2-tailed)	0.239	<0.001*	0.009*	0.080	0.040*

Statistics were made by using Pearson's correlation (significance <0.05), *statistically significant, std: standard deviation, PSM: positive surgical margins, ECD: extracapsular disease, SVI: seminal vesicles invasion, LNI: lymph nodes invasion, and BCR: biochemical recurrence.

**Table 4:** Univariate and multivariate analysis of preoperative parameters regarding the predictive potential of biochemical recurrence.

	*P *	Odds ratio	95% CI
Univariate analysis			

PSA	0.002*	1.126	1.045–1.213
PSA density	0.005*	3.408	1.454–7.987
Preoperative Gleason summary	0.233	1.214	0.882–1.671

Multivariate analysis			

PSA	0.006*	1.112	1.030–1.200
PSA density	0.009*	2.861	1.293–6.330

CI: confidence interval, *statistically significant.
